# Physician versus patient use of AI for diabetes prevention: public perceptions and comfort levels

**DOI:** 10.3389/fdgth.2026.1879415

**Published:** 2026-07-27

**Authors:** Gloria Maria Carmona Clavijo, Paige Nong, Sean Tan, Jodyn Platt

**Affiliations:** 1 TIERRA - Trust, Innovation & Ethics Research for Responsible AI, Department of Learning Health Science, Michigan Medicine, University of Michigan, Ann Arbor, MI, United States; 2 Division of Health Policy and Management, University of Minnesota School of Public Health, Minneapolis, MN, United States

**Keywords:** artificial intelligence, health systems, patient trust, primary prevention, public opinion

## Abstract

**Background:**

Type 2 diabetes is a major public health challenge that can often be prevented through lifestyle interventions. Artificial intelligence (AI) is increasingly used for risk prediction, behavioral coaching, and individualized prevention, offering scalability and low-intensity interventions. However, AI also raises ethical and regulatory concerns, especially for patient-facing tools. Limited evidence compares public comfort with physician use versus patient use for diabetes prevention.

**Methods:**

We analyzed data from a 2025 national survey conducted through the NORC AmeriSpeak Panel, a probability-based sample of 1,939 respondents. Participants evaluated two hypothetical AI use cases for diabetes prevention: Physician use of AI and patient use of AI-chatbot. Paired t-tests compared comfort across use cases. Weighted univariable and multivariable logistic regression models identified predictors of comfort.

**Results:**

Participants reported significantly greater comfort with physician use of AI than with patient use of an AI-chatbot for diabetes prevention (*p* < 0.001). Comfort across both cases was strongly associated with belief that AI benefits population health (patient-AI: OR = 3.67, *p* < 0.001; physician-AI: OR = 3.86, *p* < 0.001), trust in health system AI use (patient-AI: OR = 1.47, *p* < 0.001, physician-AI: OR = 1.72, *p* < 0.001), and physician confidence in AI reliability (patient-AI: OR = 1.31, *P* = 0.003; physician-AI: OR = 1.30, *p* < 0.001). Comfort with physician use of AI was lower among Black/African American participants than White participants (OR = 0.51, *p* < 0.001), while women reported lower comfort with patient use of AI compared than men (OR = 0.71, *p* = 0.032)

**Conclusions:**

Public comfort with AI for diabetes prevention appears higher when integrated with professional oversight. Trust in clinicians, health systems, and AI reliability may be central to acceptance. Differences across demographic groups highlight the importance of equity-focused, physician-led implementation, transparent communication, and inclusive trust-building strategies for ethical AI adoption.

## Introduction

Type 2 diabetes is a major public health challenge in the United States, affecting around 36 million adults and imposing major clinical and financial burdens (1). This often preventable condition is associated with serious health complications, including cardiovascular disease, kidney disease, neuropathy, and vision loss, as well as high healthcare costs (2). Evidence-based primary prevention strategies are aim to prevent disease onset of disease by targeting risk factors and promoting protective behaviors, such as healthy eating, physical activity, weight management, early identification of high-risk individuals, and sustainable behavior change (2). Given the prevalence of type 2 diabetes and the effectiveness of prevention strategies, primary prevention represents an opportunity for innovation (3).

AI tools are increasingly available to support risk prediction, behavioral coaching, and personalized recommendations (4). AI for primary prevention is appealing for its scalability, personalization, and capacity to deliver continuous, low-intensity interventions such as reminders and alerts (5). AI has increasingly been applied to diabetes prevention through tools that support clinicians by identifying individuals at elevated risk. These tools can also be used directly by patients to support personalized prevention strategies (6). For example, machine learning models can leverage electronic health records, demographic characteristics, laboratory values, and behavioral data to improve risk prediction. Machine learning-based and digital health interventions can provide tailored lifestyle recommendations and preventive support through chatbots or other interactive tools (7, 8). As AI technologies become more widely implemented, understanding public acceptance of AI-assisted prevention becomes important. Given that diabetes is a behavior-dependent chronic condition with strong evidence for lifestyle-based prevention and clear clinical guidelines (1, 2, 9), it provides a useful case study for studying AI integration in primary prevention.

Trust in AI, trust in clinicians, and trust in health systems (10), are likely to shape attitudes about specific uses of AI in healthcare. Understanding these factors is important for assessing patient comfort with AI tools for disease prevention (4, 11). Previous research suggests that comfort and trust in AI are influenced by multiple individual and contextual factors (12, 13) with trust and perceived value playing important roles. Drawing on technology acceptance and trust in AI frameworks, transparency may reduce uncertainty about AI-generated recommendations and thereby enhance perceived trustworthiness (12, 14, 15). Greater trust in health systems and AI tools has been associated with higher willingness to engage with such technologies (16). In parallel, perceived benefits-such as improved access, safety and effectiveness-may increase acceptance and comfort with AI use (17). Physician confidence in AI reliability can further increase patient comfort by signaling credibility and safety and influencing how AI-supported recommendations are communicated (18, 19). Finally, comfort with AI tools for preventive care may also vary across demographic and contextual factors (20), reflecting differences in experiences, expectations, and perceptions across populations.

**Figure 1:**
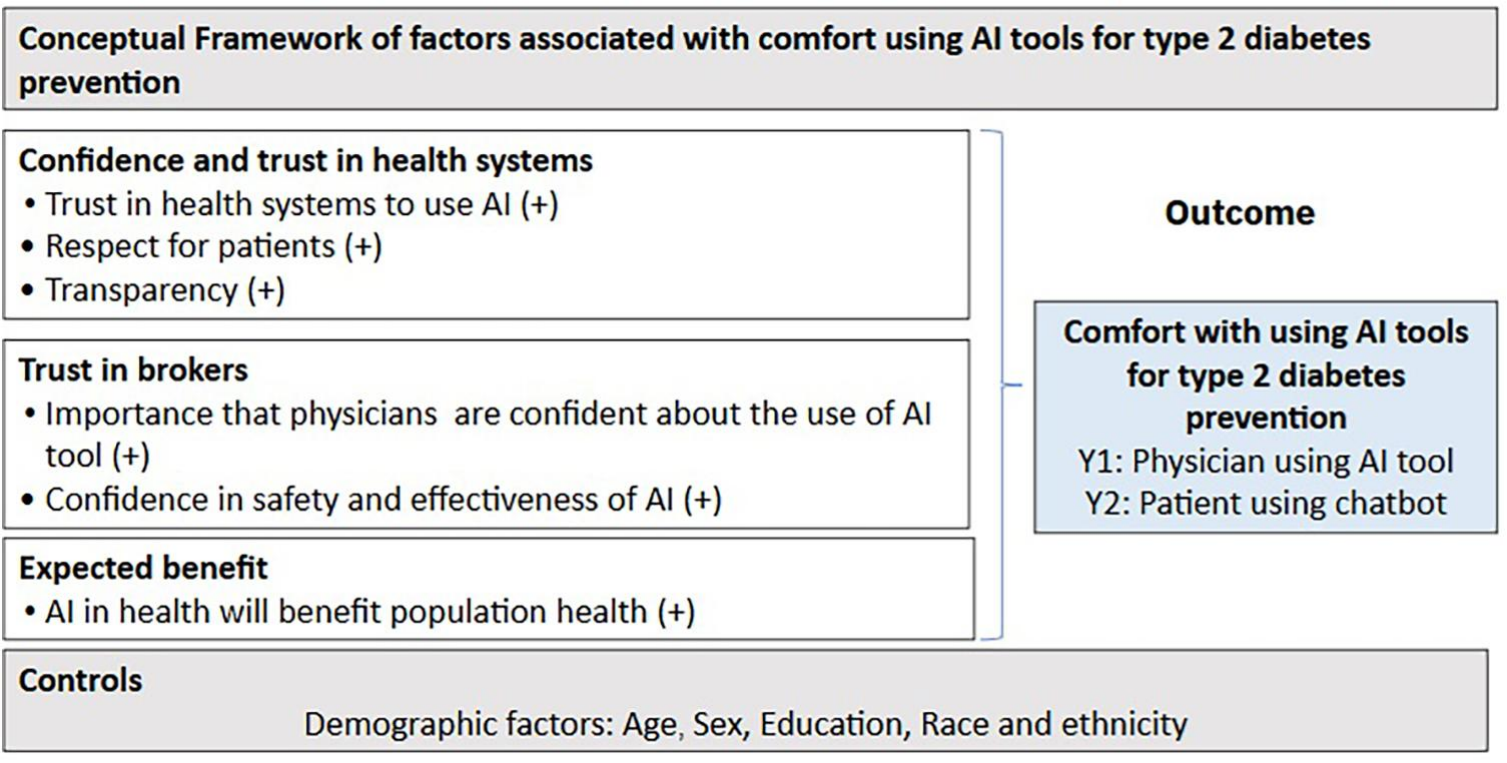
Conceptual framework of factors associated with comfort using AI tools for type 2 diabetes prevention comparing physician use of AI tool and patient using a chatbot.

In sum, whether an AI tool is used by a primary care provider to support decision making, or directly by patients for health education, its adoption is likely to be influenced by confidence and trust in health systems (i.e., beliefs that healthcare organizations use AI safely, effectively and responsibly), trust in brokers (i.e., confidence in physicians as appropriate and competent intermediaries in the use of AI and expected benefits (i.e., perceptions that AI in healthcare will improve population health outcomes). These factors are also expected to shape, and be shaped by, comfort with AI use in healthcare. Figure 1 presents the conceptual framework guiding this study and illustrates the hypothesized relationship among these factors and comfort with physician versus patient use of AI for diabetes prevention.

**Figure 2:**
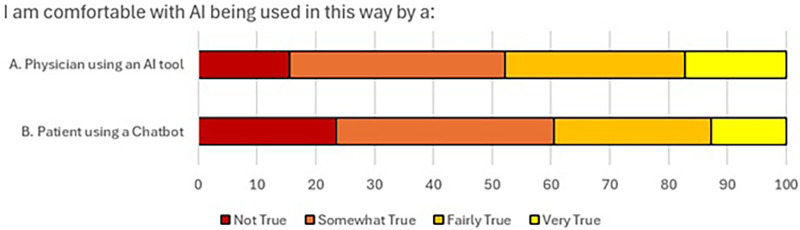
Distribution of the outcome variables. Proportion of respondents (%) reporting comfort when AI tools are used by their physician versus when the patient uses AI tools themselves to create a Type 2 Diabetes prevention plan. (*p* < 0.05) *n* = 1,939.

Patient-facing AI tools can expand reach and engagement. However, despite the growing use of AI in healthcare, little is known about public and patient comfort with these tools (12), particularly in primary prevention. Existing evidence rarely distinguishes between AI used by physicians to support prevention and AI used directly by patients, leaving gaps in understanding how comfort varies by user and context. This manuscript addresses this gap by examining the use of AI in diabetes prevention, comparing comfort levels when AI tools are used by physicians versus when patients use them independently. We also assess whether and how demographic and other predictors play a role in comfort in each context. This study explores these questions in primary prevention settings, offering insights with implications for policy, practice, and implementation science. Understanding these interactions can help providers, health systems, and policymakers determine the conditions under which AI tools for primary prevention are most acceptable, and how individual and contextual factors shape comfort with their use.

## Methods

This study used data from an original cross-sectional survey of English-speaking U.S. adults fielded in April-May 2025. The survey sample was drawn from the National Opinion Research Center's (NORC) AmeriSpeak Panel. The survey instrument was validated using cognitive testing and pilot testing prior to fielding to assess item comprehension, evaluate clarity, and identify potential sources of response error or bias. Adults aged 18 and older were considered eligible for the online survey. Black/African American respondents and, Hispanic respondents were oversampled to ensure adequate representation and statistical power. Poststratification survey weights calculated based on the Census Bureau's Current Population Survey were applied to produce nationally representative estimates.

Based on previous experiences with national surveys and focus groups (4, 21, 22), the research team produced a∼2-minute explanatory video describing how AI is used in the US healthcare system. The purpose of the video was to standardize baseline understanding of AI among respondents and reduce variability in prior knowledge. An expert advisory board convened for this study, comprised of subject matter experts, clinicians, and community members, reviewed the video and provided input on the content, definitions, and wording. The video was also presented to patient and community focus groups to clarify the contents and assess the content prior to data collection. The respondents viewed the video at the beginning of the survey. Definitions of key terms (healthcare provider, artificial intelligence, etc.) were provided and available to respondents as hover-over text each time the terms were used in the survey.

### Data and analysis

Our analytic sample consisted of respondents who provided responses to two hypothetical use cases related to the use of AI tools supporting the prevention of diabetes. All participants received both scenarios, and the order of presentation was randomized:

Case A- Physician Use of AI:
  You visit your doctor after a blood test shows that you are at-risk of developing Type 2 diabetes, a condition that results in high blood sugar levels. Managing Type 2 diabetes involves changing your diet and taking medication. **Your doctor has access to an AI tool that will create a treatment plan** of medication and diet changes for you. The AI tool uses your medical records and lab results to create the treatment plan. Your doctor reviews and updates the treatment plan before reviewing it with you.
Case B-Patient Use of AI:
  You visit your doctor after a blood test shows that you are at-risk of developing Type 2 diabetes, a condition that results in high blood sugar levels and requires changes in diet and frequent medication. Your doctor tells you about an **AI chatbot that you can use to manage your health.** The AI chatbot has access to your blood sugar data recorded by your glucose monitor and data in your electronic health record. The chatbot provides recommendations for diet and exercise routines and can answer basic health questions or suggest you contact your doctor or care team.

To answer our question of whether comfort differs between physician use and patient use of AI tools for creating a diabetes prevention plan, we asked respondents how true the following statement was for them: “I am comfortable with AI being used in this way.” Responses were captured on a scale from Not at all true (1) to Very true (4). We then conducted paired t-tests to assess whether the mean level of comfort with the use of AI tools was different from each context (Physician vs. Patients). Paired t-tests were used because parametric methods have been shown to be robust for Likert-type data in large samples (23, 24).

We generated a binary dependent variable for each context, grouping “Not true” and “Somewhat true” into one category and “Fairly true” and “Very true” into another based on the distribution of the variable.

We then examined demographic and other factors that might predict comfort with each context. Demographic factors included sex, age, race/ethnicity, and education. Additional factors included two questions assessing confidence and trust in health systems, two questions assessing trust in brokers, and one question assessing expected benefit. Table 1 lists the specific survey questions and scales used.

**Table 1:** Factors associated with comfort using AI tools for type 2 diabetes prevention.

Construct	Variable Survey question	Scale
Confidence and trust in health systems	Trust in health systems about the use of AI: “I trust my healthcare system to use AI tools responsibly”	1 = Not at all true to 4 = Very true
	Feel respected “I feel respected when I seek healthcare”.	1 = Not at all true to 4 = Very true
	Transparency “My healthcare system is transparent about how my health information is used”	1 = Not at all true to 4 = Very true
Trust in brokers	Importance that physicians are confident about the use of AI tool “It is important that the doctor is confident about the reliability of AI tools”	1 = Not at all true to 4 = Very true
	Confidence in safety and effectiveness of AI “If the AI tool meets industry standards for safety and effectiveness”	1 = Most important to 5 = Least important
Expected benefit	AI in health will benefit population health “How likely do you think it is that the use of AI in clinical decision making will improve the health of people living in the United States?”	1 = Very unlikely to 4 = Very likely

Using Stata version 19, we conducted univariate and multivariable weighted logistic regression for two different outcomes: First, we examined predictors of comfort and other relevant factors in Case A (Physician use of AI tool to create a diabetes prevention plan). Then we examined predictors of comfort with Case B (patient use of an AI tool-chatbot). Model fit was assessed in both cases using the Hosmer-Lemeshow goodness of fit test. Multicollinearity among predictors was assessed using variance inflation factors (VIF), with values below 2.5 indicating no evidence of multicollinearity.

This study was conducted in accordance with institutional ethical requirements, and informed consent was obtained from all participants. Additional details are provided in the ethics statement.

## Results

Descriptive statistics for the sample are provided in Table 2. Approximately half of the sample self-identified as female (50.8%). Respondents included those aged 18–29 years (15.6%), 30–44 years (27.8%), 45–59 years (22.7%), and over 60 years (34.9%). A similar proportion of Black-African American and Hispanic respondents participated in the survey (25% and 26%, respectively), 44% of respondents identified as White, and 5.2% identified with other racial/ethnic groups, including Asian, Pacific-Islander, multiracial, and self-identified other categories. Most respondents had at least some college or an associate's degree (61.1%).

**Table 2:** Descriptive statistics of variables used in binary logistic regression: demographic factors, independent and dependent variables (*n* = 1,939).

Variable	N (%)	Mean (SD)
Sex		
Male	955 (49.2)	–
Female	984 (50.8)	–
Age (4 categories)		
18–29	332 (15.6)	–
30–44	588 (27.8)	–
45–59	481 (22.7)	–
60+	718 (34.9)	–
Race/Ethnicity (RE4)		
White, non-Hispanic	932 (44.0)	–
Black, non-Hispanic	528 (25.0)	–
Other^a^, non-Hispanic	110 (5.2)	–
Hispanic	549 (26.0)	–
Education (EDU4)		
Less than HS/HS graduate or equivalent	502 (23.7)	–
Some college/ associates degree	848 (40.0)	–
Bachelor's degree	448 (21.1)	–
Post graduation studies/professional degree	321 (15.2)	–
Dependent Variables		
Comfort with the Physician using AI tool for a Diabetes preventive plan^b^^,^^c^		**2.49 (0.95)**
Comfort with the patient using a chatbot for a preventive Diabetes plan^b^^,^^d^		**2.27 (0.97)**
Independent Variables		
Trust in health systems about the use of AI [in general]^b^		2.19 (0.93)
Feel respected^b^		2.82 (0.92)
Transparency^b^		2.20 (0.89)
It is important that the doctor is confident about the reliability of AI [in general]^b^		3.10 (1.04)
Safe and effectiveness of AI^g^		2.52 (1.24)
AI in health will benefit population health^e^^,^^f^		2.09 (0.76)

Bold values are the study outcomes.

a Includes Asian-Pacific Islander (*n* = 57), multi-racial (*n* = 34), and self-identified others (*n* = 15).

b Range: 1 = Not true 2 =Somewhat true 3 =Fairly true 4 =Very true.

c Recoded as Binary variable in logistic regression analyses, coded as follows: (0**)** Not true (15.7%) or somewhat true (36.7%); (1) Fairly true (30.3%) or very true (17.1%).

d Recoded as Binary variable in logistic regression analyses, coded as follows: (0**)** Not true (24.2%) or somewhat true (37.1%); (1) Fairly true (26.1%) or very true (12.6%).

e Range: 1 = Very unlikely 2 = Unlikely 3 = Likely 4 = Very likely.

f Recoded as Binary variable in logistic regression analyses, coded as follow: (0) Very unlikely (8.6%) or Unlikely (34%), (1) Likely (49.35%) or very likely (8%).

g 1= Most important - 5 = least important.

Most people felt that it is important that their doctor is confident about the reliability of an AI tool in general (mean 3.1, SD = 1.04), and the mean score for feeling respected when seeking medical care was 2.8 (SD = 0.92). Overall, trust in health systems to use AI responsibly and to be transparent about the use of AI was low (mean 2.2, SD = 0.93, and mean 2.2, SD = 0.89, respectively). People felt that the use of AI in health is likely to benefit the population health (mean 2.1 SD = 0.76). The mean of importance regarding the statement that an AI tool should meet industry standards for safety and effectiveness of AI was 2.5 (SD = 1.24).

**Comfort with the use of AI by physician versus patients of AI for a diabetes prevention plan.**

The mean score of the first dependent variable, whether people feel comfortable with the physician using an AI tool for a diabetes preventive plan was 2.49 (SD = 0.95). The second dependent variable measured whether people feel comfortable with a patient using a chatbot for a diabetes prevention plan and the mean was 2.27 (SD = 0.97). We found that the difference in mean comfort between physician use and patient use of AI was statistically significant based on paired t-test (*p* < 0.001). Although comfort was statistically higher for physician use of AI, the mean difference was modest (0.21 points on a 4-point scale), suggesting that physician involvement improves comfort but may not fully determine acceptance.

**Univariable analysis: Physicians use of AI versus patients of AI for a diabetes prevention plan.**

When examining the univariable relationship using weighted logistic models (Tables 3, 4), we found that women were less likely than men to report comfort with patient use of AI (OR = 0.51 *p* = 0.001). For physician use of AI, comfort did not differ significantly by sex (*p* > 0.05).

**Table 3:** Unweighted and weighted binary logistic regression analysis of comfortability with the **patient** using a chatbot for a diabetes prevention plan: univariable and multivariable analysis. (*n* = 1,939).

Patient using Chatbot [I am comfortable with AI being used in this way]								
	Unweighted				Weighted			
	Univariable		Multivariable		Univariable		Multivariable	
Variable	OR	*p*-value	OR	*p*-value	OR	*p*-value	OR	*p*-value
Sex								
Male	ref	ref	ref	ref	ref	ref	ref	ref
Female	**0.63**	<**0.001**	** 0.68**	**0.001**	**0.64**	**0.001**	**0.71**	**0.032**
Age								
18–29	ref	ref	ref	ref	ref	ref	ref	ref
30–44	0.88	0.352	0.69	**0.037**	0.89	0.571	0.78	0.290
45–59	0.82	0.185	0.71	0.066	0.92	0.710	0.87	0.604
60+	0.86	0.267	0.54	**0.001**	0.96	0.834	0.63	0.065
Educ								
Less than HS/HS graduate or equivalent	ref	ref	Ref	ref	ref	ref	ref	ref
Some college	0.90	0.373	0.92	0.574	0.92	0.606	0.85	0.437
Bachelor	1.13	0.354	1.06	0.754	1.21	0.307	1.06	0.98
Postgraduation studies	1.31	0.060	1.33	0.139	1.24	0.282	1.03	0.912
Race/ethnicity								
White	ref	ref	Ref	ref	ref	ref	Ref	ref
Black Americans	1.06	0.627	1.13	**0.037**	1.11	0.472	1.29	0.157
Others	1.44	0.073	1.40	0.066	1.60	0.098	1.74	0.071
Hispanics	1.07	0.546	1.37	**0.001**	1.03	0.830	1.25	0.252
Trust in health systems about AI use	**2.64**	<**0.001**	** 1.77**	<**0.001**	**2.47**	**0.001**	**1.47**	<**0.001**
Feel respected	**1.51**	<**0.001**	1.11	0.115	**1.58**	<**0.001**	1.17	0.100
Transparency	**1.64**	<**0.001**	** 1.16**	**0.036**	**1.73**	<**0.001**	**1.24**	**0.029**
Physician confidence in the reliability of AI	**1.63**	<**0.001**	** 1.31**	<**0.001**	**1.63**	**0.001**	**1.31**	**0.003**
Safe and effectiveness of AI	**0**.**87**	<**0**.**001**	** 0**.**89**	**0**.**004**	**0**.**86**	**0**.**008**	0.96	0.462
AI effect on population health	**1**.**69**	<**0**.**001**	**2**.**99**	<**0**.**001**	**1**.**78**	<**0**.**001**	**3**.**67**	<**0**.**001**

Bold values are the ORs results of the predictors with significant value.

Significance codes: *p* < 0.05.

**Table 4:** Unweighted and weighted binary logistic regression analysis of comfortability with the **physician** using a tool for a diabetes prevention plan: univariable and multivariable analysis.

Physician using a tool [I am comfortable with AI being used in this way]								
	Unweighted				Weighted			
	Univariable		Multivariable		Univariable		Multivariable	
Variable	OR	*p*-value	OR	*p*-value	OR	*p*-value	OR	*p*-value
Sex								
Male	ref	ref	Ref	ref	Ref	ref	Ref	Ref
Female	**0.72**	<**0.001**	0.85	0.134	**0.69**	**0.004**	0.058	0.546
Age								
18–29	ref	ref	Ref	ref	Ref	ref	Ref	Ref
30–44	**1.44**	**0.009**	1.22	0.268	**1.49**	**0.048**	1.35	0.218
45–59	**1.62**	**0.001**	1.43	0.059	**1.57**	**0.038**	1.35	0.262
60+	**1.98**	<**0.001**	1.24	0.226	**2.32**	<**0.001**	1.40	0.176
Educ								
Less than HS/HS graduate or equivalent	ref	ref	Ref	ref	Ref	ref	Ref	Ref
Some college	1.20	0.114	1.09	0.547	1.28	0.124	1.06	0.778
Bachelor	**1.70**	<**0.001**	1.27	0.161	**1.95**	<**0.001**	1.43	0.126
Postgrad	**1.87**	<**0.001**	**1.47**	**0.044**	**1.85**	**0.002**	1.50	0.112
Race/ethnicity								
White	ref	ref	Ref	ref	Ref	ref	Ref	Ref
Black Americans	**0.59**	<**0.001**	**0.56**	<**0.001**	**0.58**	<**0.001**	**0.51**	<**0.001**
Others	0.82	0.312	0.71	0.174	0.84	0.538	0.67	0.207
Hispanics	**0.70**	**0.001**	0.88	0.362	**0.62**	**0.002**	1.39	0.176
Trust in health systems about the use of AI	**2.54**	<**0.001**	**1.66**	<**0.001**	**2.75**	<**0.001**	**1.72**	<**0.001**
Feel respected	**1.60**	<**0.001**	**1.25**	**0.001**	**1.67**	<**0.001**	**1.23**	**0.034**
Transparency	1.48	<**0.001**	0.96	0.952	**1.65**	<**0.001**	1.22	0.080
Physician confidence in the reliability of AI	**1.87**	<**0.001**	**1.39**	<**0.001**	**2.03**	<**0.001**	**1.30**	<**0.001**
Safe and effectiveness of AI	**0.84**	<**0.001**	0.96	0.325	0.31	0.072	1.10	0.152
AI effect on population health	**1.81**	<**0.001**	**3.52**	<**0.001**	**1.94**	<**0.001**	**3.86**	<**0.001**

Bold values are the ORs results of the predictors with significant value.

Significance codes: *p* < 0.05.

Education was not significantly associated with comfort for patient use of AI (*p* > 0.05). However, for physician use of AI, respondents with a bachelor's degree (OR = 1.95, *p* < 0.001) or postgraduate education (OR = 1.85, *p* = 0.002) were more likely to report comfort compared with those with less education.

Race/ethnicity was not significantly associated with comfort for patient use of AI (*p* > 0.05). For physician use of AI, Black/African American (OR = 0.58, *p* < 0.001) and Hispanic respondents (OR = 0.62, *p* = 0.002) were less likely to report comfort compared with White respondents.

Belief that AI would benefit the population was positively associated with comfort for patient use of AI (OR = 1.78, *p* < 0.001) and positively associated with comfort with physician use of AI (OR = 1.94, *p* < 0.001) in weighted univariable analyses. Trust in health systems, confidence in a physician's use of AI, perceived transparency, and feeling respected were all positively associated with comfort across both models. Comfort with patient use of AI was lower among respondents with greater concern about AI safety and effectiveness (OR = 0.86, *p* = 0.008). For physician use of AI, this concern was not significantly associated with comfort (*p* > 0.05).

**Multivariable analysis: AI use by physicians versus patients for a diabetes prevention plan.**

In the weighted multivariable logistic regression model including comfort with both cases and the independent variables (Tables 3, 4), we found that women were less likely than men to report comfort with patient use of AI (OR = 0.71, *p* = 0.032). For physician use of AI, comfort did not differ significantly by sex (*p* > 0.05). Age and education were not significantly associated with comfort for either patient or physician use of AI (*p* > 0.05).

Race/ethnicity was not significantly associated with comfort for patient use of AI (*p* > 0.05). For physician use of AI, Black/African American respondents remained significantly less likely to report comfort (OR = 0.51, *p* < 0.001).

Belief that AI would benefit the population health was positively associated with comfort for patient use of AI (OR = 3.67, *p* < 0.001) and physician use of AI (OR = 3.86, *p* < 0.001). Trust in the health system's use of AI was positively associated with comfort for patient use of AI (OR = 1.47, *p* < 0.001) and physician use of AI (OR = 1.72, *p* < 0.001). Confidence in physician's use of AI was also positively associated with comfort for patient use of AI and (OR = 1.31, *p* = 0.003) and physicians' use of AI (OR = 1.30, *p* < 0.001). Feeling respected was positively associated with comfort for physician use of AI (OR = 1.23, *p* = 0.034). In the case of patient use of AI, feeling respected was not significantly associated with comfort for patient use of AI (OR = 1.17, *p* = 0.10).

Variance inflation factors ranged from 1.03 to 2.42 (mean VIF = 1.50), indicating no evidence of multicollinearity among predictors. Models indicated acceptable fit for both logistic regression models. The Hosmer-Lemeshow goodness of fit test showed no evidence of poor fit for the patient chatbot case [x2 (8) = 6.30, *p* = 0.61] or the physician-use case [x2 (8) = 12.62, *p* = 0.13].

In addition to the statistical significance, the magnitude of associations varied across predictors. The strongest associations with comfort were observed for belief that AI benefits population health (patient use OR = 3.67; physician use OR = 3.86), indicating substantially higher odds of comfort among respondents who perceived greater population-level benefits of AI. Other predictors, including trust in health systems and confidence in physician AI use, showed more modest but consistent associations with comfort.

Demographic differences highlight important equity considerations, including lower comfort with physician use of AI among Black/African American respondents and lower comfort with patient use of AI among women.

## Discussion

This study examined patient comfort with AI tools for Type 2 diabetes prevention and found greater comfort with physician use of AI compared with patient use of AI tools. Participants reported significantly higher comfort with physician-led AI, and comfort with both approaches was strongly associated with beliefs in the benefits of AI, trust in the healthcare system, confidence in provider use of AI and feeling respected in care. However, differences emerged across groups: women were less comfortable with patient use of AI and Black/African American respondents were less comfortable with physician use of AI. While physician-led AI approaches were viewed more favorably in our study, these should not be interpreted as evidence that physician-led models are preferred in real world implementation. Our findings, however, highlight the likely importance of trust, physician involvement, and equity in shaping public comfort with AI and suggest that there may be important distinctions to be made for different applications and users.

Our findings are consistent with prior studies emphasizing the importance of physician involvement in AI-supported care (25). In AI-assisted diabetic retinopathy screening, patients supported AI use but preferred that clinicians review and explain results (26). Similarly, Blease et al. found that clinicians expressed concerns about AI tools providing advice without physician oversight, emphasizing the importance of human involvement in clinical decision-making (27). Collectively, this evidence reinforces our findings that physician supervision enhances perceptions of safety, accountability, and proper interpretation of AI recommendations.

Lower comfort with patient-managed AI tools, such as chatbots, is also consistent with previous evidence. Although patients recognize the potential of AI to improve access to health information, concerns persist regarding accuracy, privacy, and lack of professional oversight (28). In the diabetes-specific context, patients value AI tools for education and self-management support but also express a strong need for information, safety, and trust in these tools, suggesting caution about relying on AI without supportive guidance (29). These concerns likely reflect the lower comfort with patient-led prevention strategies observed in our study.

More broadly, our results show that acceptance of AI in healthcare is closely linked to trust in physicians and the healthcare system. Studies in radiology and chronic disease show that patients are more willing to accept AI when integrated into physician-led care rather than used independently (30). These patterns suggest that, in the context of hypothetical diabetes prevention scenarios, embedding AI within physician-supervised AI programs may provide reassurance through clinical accountability, contextual interpretation, and professional responsibility. Future research should examine whether these preferences translate into actual adoption and sustained use of AI tools.

Importantly, our results also indicated greater comfort for physician-led AI prevention tools, except for Black/African American adults. Historical and structural factors, including well-documented medical distrust among minoritized populations (31), as well as evidence of racial bias and unequal performance in clinical AI systems (32), may shape mistrust and skepticism in providers and emerging AI tools, particularly those used and controlled by physicians. Physician involvement alone is not likely to fully address underlying concerns about trust, bias, and unequal treatment. Clinical AI systems are susceptible to reproducing or amplifying existing inequities through differential performance across population groups (32, 33). Studies of algorithmic decision-making note that concerns about fairness, transparency, and accuracy are central to trust and acceptance of AI in healthcare (34, 35); our findings suggest these characteristics may be necessary but not sufficient given historical and contemporary inequity in healthcare.

Similarly, women reported lower comfort with patient-led AI prevention tools compared with men. This finding suggests that women may have distinct concerns about using patient-led AI tools, potentially reflecting differences in trust, perceived risk, or expectations of support in healthcare decision-making, and is consistent with prior studies that have found differences by sex in trust and comfort (36–38). Emerging work also suggests gendered differences in AI perceptions, with women reporting higher perceived risk and greater caution toward AI systems, which may reflect lower acceptance of patient-led AI tools (37). Additionally, these differences should be explored further. Studies conducted in human-computer interaction and psychology research have shown that technology adoption is shaped by perceived usefulness, perceived risk and trust in automation, which can vary by gender (36, 38). If AI prevention tools are adopted widely, but remain less acceptable to populations that experience health disparities and minoritization, these technologies could unintentionally reflect to widening disparities in prevention, and disease management outcomes. These results underscore the importance of tailoring AI deployment strategies to address specific trust, safety, effectiveness, and transparency concerns of diverse patient groups.

Demonstrating benefits, trust in brokers (i.e., those charged with ensuring quality), and confidence in health systems emerged as key factors associated with comfort in both models. Specifically, we found that people who valued transparency of health systems were more likely to be comfortable with the patient use case and feeling respected was positively associated with comfort across both models. These findings suggest investing in the trustworthiness of health systems, the quality of relationships between clinicians and patients, ensuring transparency, and demonstrating the benefit of AI will be important to patients.

There are limitations to this study that should inform interpretation. Survey responses were based on hypothetical scenarios and may not fully generalize attitudes toward other emerging disease prevention tools. Further research should explore additional clinical use cases to assess the broader applicability of these findings. While the video was designed to be neutral and informational, we acknowledge that any standardized framing may still have influenced respondents' baseline perceptions. This study did not assess AI familiarity, digital literacy, diabetes diagnosis, personal or family history of diabetes, which may influence perceptions of AI and should be examined in future studies. Additionally, our study is limited to inferences about association and not causation, which points to a need for future longitudinal analysis. This is particularly important given the pace with which the use of AI tools for disease prevention is evolving for physicians and patients. Future research should examine real-world applications of physician use of AI versus patient use of AI prevention tools and explore how trust, comfort, and use evolve over time. Qualitative studies exploring why individuals feel comfortable or concerned about different models of who leads AI prevention tools would provide important context for these findings. At the same time, our study provides a baseline understanding of public attitudes toward AI tools for diabetes prevention and underscores the importance of trust in shaping acceptance of AI within preventive care.

## Conclusion

The greater public comfort with physician use of AI compared with patient use of AI tools for Type 2 diabetes prevention, highlighting the central role of trust in physicians and the healthcare system. Comfort with both approaches was strongly associated with trust in the healthcare system, confidence in physicians' use of AI tools, and beliefs about the benefit of AI for population health. However, women were less comfortable with patient use of AI, and Black/African American respondents were less comfortable with physician use of AI, highlighting important differences across populations. These findings should be interpreted cautiously given that they are based on hypothetical scenarios and reflect perceived comfort rather than actual use or behavior. Future research should examine real-world implementation to assess how these preferences translate into practice. These findings suggest that clinician involvement, trust, and attention to equity will be critical for supporting patient acceptance and ensuring the safe and equitable implementation of AI in diabetes prevention. These findings could guide health system decisions, regulatory approaches, and the design of patient-versus clinician-facing tools, supporting the fair and acceptable implementation of AI.

## Data Availability

The raw data supporting the conclusions of this article will be made available by the authors, without undue reservation.
